# Theoretical Study of the Adsorption and Sensing Properties of Cr-Doped SnP_3_ Monolayer for Dissolved Characteristic Gases in Oil

**DOI:** 10.3390/ma17194812

**Published:** 2024-09-30

**Authors:** Chengjiang Wang, Xiangjia Liu, Feiyang Xie, Xuze Wang, Pengdi Zhang

**Affiliations:** College of Electrical Engineering & New Energy, China Three Gorges University, Yichang 443002, China; cj-wang@ctgu.edu.cn (C.W.); 202308580121257@ctgu.edu.cn (F.X.); 202308580121229@ctgu.edu.cn (X.W.); 202308580121314@ctgu.edu.cn (P.Z.)

**Keywords:** dissolved gases in oil, Cr-SnP_3_ monolayer, gas adsorption, the first principle theory, adsorption performance

## Abstract

Dissolved gas analysis (DGA) is a vital method for the online detection of transformer operation state. The adsorption performance of a SnP_3_ monolayer modified by transition metal Cr regarding six characteristic gases (CO, C_2_H_4_, C_2_H_2_, CH_4_, H_2_, C_2_H_6_) dissolved in oil was studied. The study reveals the relevant adsorption and gas-sensing response mechanisms through calculations of the adsorption energy, density of states, differential charge density, energy gap, and recovery time. The results display a considerable increase in the adsorption effect of the Cr-SnP_3_ monolayer on six gases. The CO, C_2_H_2_, and C_2_H_4_ gases lead to chemical adsorption, and the CH_4_, H_2_, and C_2_H_6_ gases lead to physical adsorption. Combined with the recovery time, the Cr-SnP_3_ monolayer has a strong adsorption effect on CO and C_2_H_2_ gases at normal temperatures and even high temperatures, and the adsorption is stable. C_2_H_4_ gas can be rapidly desorbed from the Cr-SnP_3_ monolayer at 398 K. Therefore, the Cr-SnP_3_ monolayer can be expected to serve as a CO and C_2_H_2_ gas adsorbent and a resistive gas sensor for C_2_H_4_ gas. This research offers a theoretical foundation for the development of the Cr-SnP_3_ monolayer in gas-sensitive materials.

## 1. Introduction

The transformer is the primary component of machinery in a power system; it performs the crucial functions of power transfer, distribution, and the conversion of voltage [1]. The transformer’s regular operation is crucial to the electrical system’s security, reliable operation, and power supply. Over 90% of all transformers are oil-immersed power transformers [2]. Transformer oil is a high-alkane mixture. Under long-term operation, due to pollution, high temperatures, design defects, ageing, and other reasons, hydrocarbons in transformer oil often crack and decompose into CO, C_2_H_2_, C_2_H_4,_ CH_4_, C_2_H_4_, H_2_, C_2_H_6_, and other gases. These gases are often dissolved in transformer oil, so they are called dissolved fault characteristic gases in transformer oil [3]. Transformer fault types are significantly correlated with the components of the dissolved gas in oil. Therefore, by means of the sensitive identification of dissolved gas components in transformer oil, the internal fault situation and development trends in the transformer can be effectively understood, and then the insulation operation state of the transformer can be scientifically evaluated, which can effectively reduce the incidence of insulation accidents [4].

Two-dimensional (2D) materials have emerged as a popular area of study in the scientific community [5]. The distinctive structural and electrical characteristics of phosphorene and black phosphorene have garnered significant interest [6]. Among them, the phosphides of IV-V compounds, such as SnP_3_ [7] and GeP_3_ [8], have demonstrated exceptional promise in terms of their optical and electrical qualities [9]. Research in this field provides new possibilities for exploring the potential applications of new materials. The SnP_3_ monolayer, a two-dimensional substance, is a layered compound with a triangular space group [10], and has a honeycomb planar structure. Gas molecules are easily adsorbed onto SnP_3_ due to its large specific surface area. SnP_3_ exhibits semiconductor properties in a single-layer structure. The band gap values obtained with the PBE and HSE06 functions are 0.53 eV and 0.82 eV, respectively [11]. In addition, the SnP_3_ monolayer has high carrier mobility [12], and is reportedly a promising material for small gas sensing [13]. Although the intrinsic SnP_3_ monolayer has a limited adsorption capacity, its electronic properties can be improved using doping metals [14]. This helps to improve the sensitivity and sensor response of the SnP_3_ monolayer [13]. Recently, Cr has attracted increasing attention as a cheap and promising metal catalyst [15]. For example, some scholars have found that 3D transition metal Cr-doped graphene can efficiently adsorb C_2_H_2_, and the adsorption energy can reach 1.44 eV [16]. Some experts have synthesised Cr-doped SnO_2_ nanowires via vapour deposition and found that doping with 1.8% Cr (atomic fraction) can increase the oxygen gas-sensing performance of SnO_2_ nanowires by 28% at 250 °C [17]. The doping adsorption of SnP_3_ monolayers is less studied, particularly the gas-sensing properties after doping with noble metals [6].

Therefore, this paper proposes a modification to the doping of single-layer SnP_3_ with transition metal Cr. Firstly, the most stable doping structure (Cr-SnP_3_) was established. After this, various gas adsorption structures were built. Through geometric optimisation, the most stable Cr-SnP_3_ monolayer structure for the absorption of the six gases was determined. Finally, the adsorption properties of Cr-SnP_3_ for six gases (CO, C_2_H_2_, C_2_H_4_, CH_4_, H_2_, and C_2_H_6_) were investigated by analysing the adsorption energy, charge transfer, density of states, differential charge density, energy gap, and recovery time. This finding suggests the prospect of employing Cr-SnP_3_ as an absorption material for gases in the transformer oil in gas sensors.

## 2. Calculation Method and Structural Model

### 2.1. Parameter Setting

The adsorption behaviour of six gases (CO, C_2_H_2_, C_2_H_4_, CH_4_, H_2_, and C_2_H_6_) on a Cr-SnP_3_ monolayer was simulated. The establishment of all models and the calculations were completed using the DMol3 module of Materials Studio. The optimisation, energy, and related properties of the structure were computed using the PBE. The DNP was selected as the basis set function of the linear combination of atomic orbitals, and the Grimme method in DFT-D dispersion correction was used to correct the influence of the van der Waals force [18]. The gas molecules and transition metals used the all-electron model and DSPP treatments, respectively [19]. The convergence accuracy of SCF was 1.0 × 10^−6^, and the DIIS value was set to 6. The Smearing value was 0.005 Ha [20]. The whole system may have magnetic properties because Cr is a transition metal. Therefore, the electron spin was included in the calculation to ensure accuracy.

In this paper, we established a 3 × 3 × 1 single-layer SnP_3_ supercell periodic structure which contained 54 P and 18 Sn atoms. The vacuum layer was 20Å. The optimized lattice parameters of the SnP_3_ monolayer were a = b = 7.42Å, c = 10.28Å, which are almost consistent with the experimental values (a = b = 7.38Å, c = 10.51Å) [6]. The accuracy of the model was proved.

**Figure 1:**
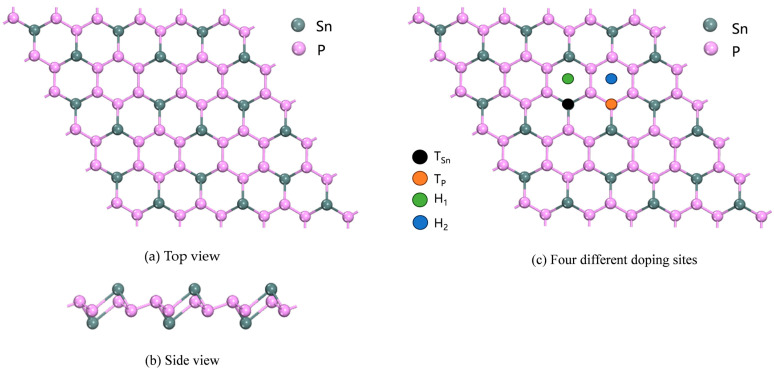
Intrinsic SnP_3_ monolayer and four different doping sites.

The intrinsic SnP_3_ monolayer was established, and different sites were doped in the folded honeycomb repeat layer. The structure of the intrinsic SnP_3_ and four cases are shown in Figure 1; the cases are T_Sn_ (above the Sn atom), T_P_ (above the P atom), H_1_ (above the centre of the hexagonal ring composed of P and Sn), and H_2_ (above the centre of the hexagonal ring composed of P). Finally, the energy of each system after doping was calculated.

The binding energies *E_b_* of different dopant sites can be compared to determine the optimal doped structure, which is described by Equation (1)

(1) $$E_{b}=E_{\left(Cr\text{-}SnP_{3}\right)}-E_{\left(SnP_{3}\right)}-E_{Cr}$$

where $E_{\left(Cr\text{-}SnP_{3}\right)}$ is the energy of the system after the Cr doping of SnP_3_; $E_{\left(SnP_{3}\right)}$ and $E_{Cr}$ are the energies of intrinsic SnP_3_ and Cr atoms, respectively.

The adsorption energy *E_ads_* was applied to assess the stability of the adsorption system, which is defined by Equation (2)

(2) $$E_{ads}=E_{\left(gas/Cr\text{-}SnP_{3}\right)}-E_{\left(Cr\text{-}SnP_{3}\right)}-E_{gas}$$

where $E_{\left(gas/Cr\text{-}SnP_{3}\right)}$ is the energy of gas/Cr-SnP_3_; $E_{gas}$ is the energy of the gas molecules.

The charge transfer *Q_t_* between the gas molecules and Cr-SnP_3_ material is calculated by Equation (3)

(3) $$Q_{t}=Q_{a}-Q_{b}$$

where *Q_a_* and *Q_b_* denote the net charge of the gas molecules after and before adsorption [21].

### 2.2. Dissolved Characteristic Gas in Oil

**Figure 2:**
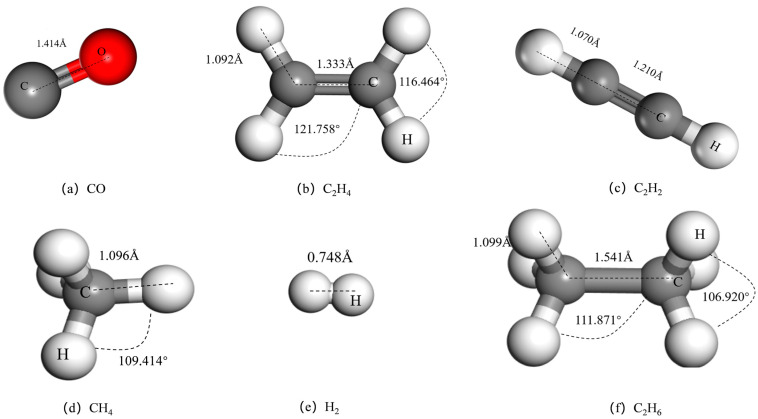
Structures of (**a**) CO, (**b**) C_2_H_4_, (**c**) C_2_H_2_, (**d**) CH_4_, (**e**) H_2_, and (**f**) C_2_H_6_ molecules.

The optimized structure models of dissolved characteristic gases (CO, C_2_H_4_, C_2_H_2_, CH_4_, H_2_, and C_2_H_6_) in oil are shown in Figure 2.

### 2.3. Optimised Structural Model of Cr-SnP_3_

The four structural parameters of Cr-doped SnP_3_ are shown in Table 1. Through a comparison of the absolute values of binding energy, T_Sn_ was determined to be the best doping site.

Chemical hardness (*η*) is an indicator of chemical stability. A higher chemical hardness indicates that the stability of the system is improved [22]. The η is calculated using Equation (4).

(4) $$\eta =(E_{HOMO}-E_{LUMO})/2$$

**Figure 3:**
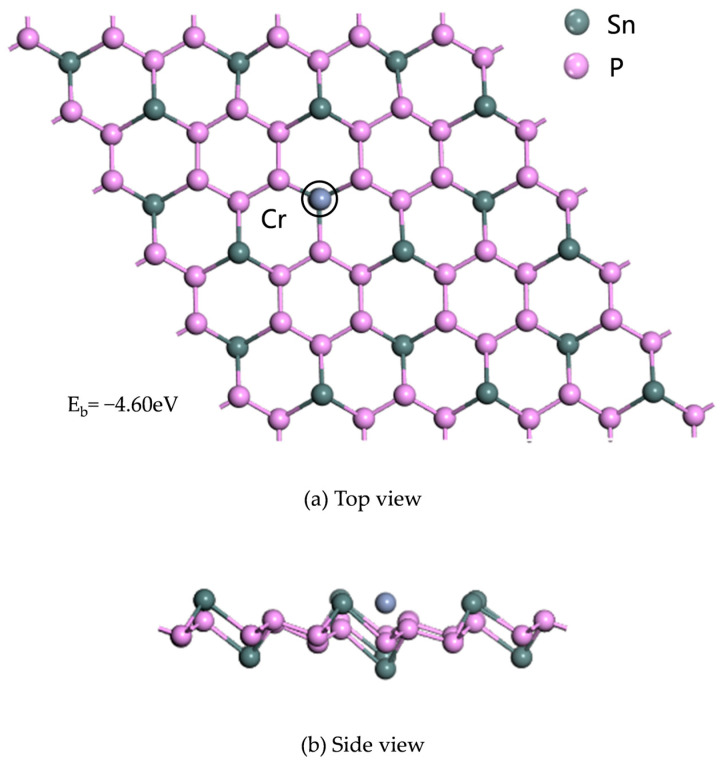
(**a**) Top view and (**b**) side view of the optimal doping structure of Cr-SnP_3_.

The chemical hardness of the four doping sites is shown in Table 1. T_sn_ corresponds to the maximum chemical hardness, indicating that the system is the most stable. This is consistent with the E_b_ analysis. The optimal structural model of Cr-SnP_3_ is shown in Figure 3.

### 2.4. The Density of States of the Cr-SnP_3_ Monolayer

**Figure 4:**
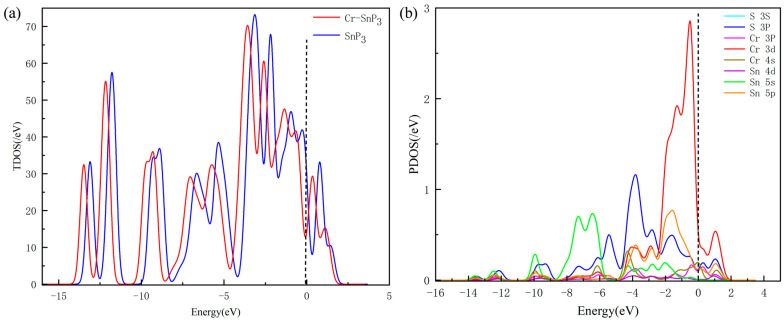
(**a**) TDOS of intrinsic SnP_3_ and Cr-SnP_3_ monolayer; (**b**) PDOS of Cr-SnP_3_ monolayer.

In order to illustrate the difference in the electronic structure of the SnP_3_ crystal plane before and after Cr doping, the density of states (DOS) curves were analysed, as depicted in Figure 4.

The dotted line at 0 eV in Figure 4a represents the Fermi level. The total density of states (TDOS) of Cr-SnP_3_ follows a lower-energy path to the left, the shape changes slightly, and the valence band is filled with more electrons. However, the peak value of the TDOS before and after doping is similar, and the overall density of states is still composed of three parts—the conduction band and upper and lower valence bands—which is similar to the density of states of the intrinsic SnP_3_. This demonstrates that the crystal energy of SnP_3_ is altered by the addition of Cr atoms, but the crystal structure does not change.

The local density of states of Cr-SnP_3_ in Figure 4b shows that S-3P, Cr-3d, and Sn-5p have obvious overlapping peaks at −15~5 eV, which suggests that the orbitals are strongly hybridising. As a result, it may be deduced that the Cr atom and SnP_3_ can form a stable doping structure.

## 3. Analysis of Adsorption Results

### 3.1. Investigation of the Adsorption Properties of Cr-SnP_3_ on CO, C_2_H_4_, C_2_H_2_, CH_4_, H_2_, and C_2_H_6_

**Figure 5:**
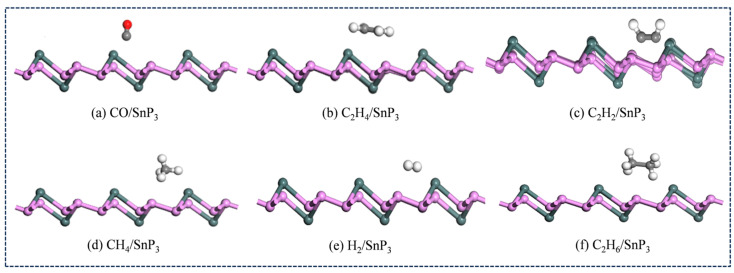
The optimal adsorption model of (**a**) CO, (**b**) C_2_H_4_, (**c**) C_2_H_2_, (**d**) CH_4_, (**e**) H_2_, and (**f**) C_2_H_6_ molecules on an intrinsic SnP_3_ monolayer.

**Figure 6:**
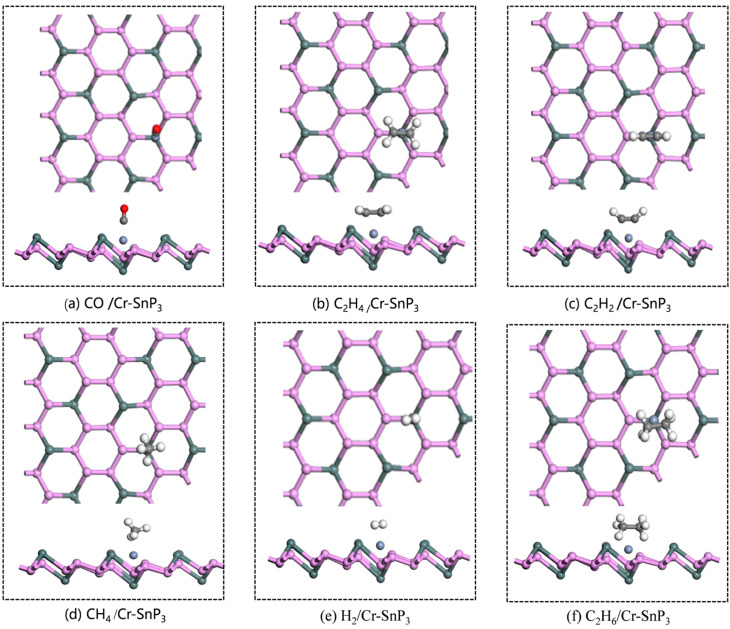
The optimal adsorption model of (**a**) CO, (**b**) C_2_H_4_, (**c**) C_2_H_2_, (**d**) CH_4_, (**e**) H_2_, and (**f**) C_2_H_6_ molecules on the Cr-SnP_3_ monolayer.

Since the gas molecules are placed on the Cr-SnP_3_ monolayer in different ways, the article established a variety of gas-monolayer adsorption systems and optimized the different positions so as to obtain the lowest energy-adsorption system. It was finally determined that the most stable adsorption configurations are achieved when gas molecules are adsorbed above the Cr atom. The optimal adsorption models of intrinsic SnP_3_ and Cr-SnP_3_ for six gases are shown in Figure 5 and Figure 6, respectively. The adsorption parameters are listed in Table 2.

In Table 2, the adsorption energy of the six gases is negative, which shows that the adsorption behaviour is a spontaneous exothermic reaction. The E_ads_ of intrinsic SnP_3_ to CO, C_2_H_4_, CH_4_, H_2_, and C_2_H_6_ is relatively small, the charge transfer amount is very weak, and the adsorption distance is far. It is impossible for the five gases to be stably adsorbed on the surface of intrinsic SnP_3_. After the modification of SnP_3_ by Cr metal, the adsorption parameters increased significantly, and the overall adsorption effect was ideal. The adsorption energies of CO, C_2_H_4_, and C_2_H_2_ on Cr-SnP_3_ were −1.643, −1.156, and −2.129 eV, respectively, and the corresponding adsorption distances were 1.939, 2.070, and 1.896 Å, respectively. The H-C-C bond angle in C_2_H_2_ changed from 180° to 142.09°, and the C_2_H_4_’s H-C-C bond was slightly inclined upward during the surface reaction. It was preliminarily judged that the adsorption of CO, C_2_H_4_, and C_2_H_2_ molecules by the Cr-SnP_3_ monolayer belonged to chemical adsorption. Generally speaking, the absolute value of the adsorption energy above 0.8 eV was for chemical adsorption. The adsorption energy of CH_4_, H_2_, and C_2_H_6_ gases did not reach −0.8 eV, and the adsorption distance was far, with no significant change observed in the bond lengths. Therefore, the three gases’ adsorptions were likely to be physical adsorption.

### 3.2. Density of State

**Figure 7:**
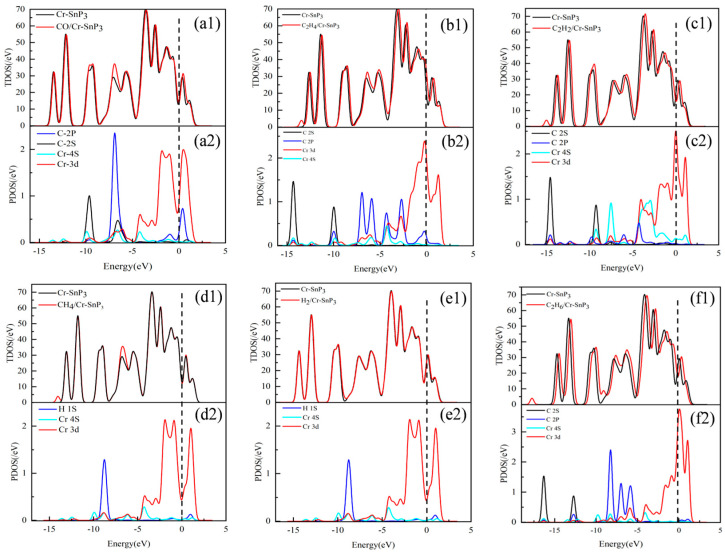
TDOS and PDOS of (**a1**,**a2**) CO, (**b1**,**b2**) C_2_H_4_, (**c1**,**c2**) C_2_H_2_, (**d1**,**d2**) CH_4_, (**e1**,**e2**) H_2_, and (**f1**,**f2**) C_2_H_6_ adsorption systems.

In this paper, the total density of states (TDOS) and partial density of states (PDOS) of six gas-adsorption systems were studied, as shown in Figure 7.

Figure 7(a1,b1,c1) shows that the TDOS curves slightly shifted to the right after the adsorption of CO, C_2_H_4_, and C_2_H_2_ gases by Cr-SnP_3_. The PDOS of the three gases shows that this was due to the introduction of gas molecules and that the main contribution was from the C-2P orbitals. The C-2S, C-2P, Cr-3d, and Cr-4S orbitals formed a wide range of orbital overlaps at multiple energy levels in the −15 eV~5 eV interval with multiple overlapping peaks, indicating the existence of interatomic orbital hybridization. This confirms that the interaction between the two is chemical adsorption.

Figure 7d,e shows the curves of the CH_4_ and H_2_ adsorption systems, respectively. The TDOS curves of Cr-SnP_3_ before and after the adsorption of gas almost completely overlap, and the peak value does not change significantly. By observing Figure 7(d2,e2) PDOS, it can be seen that there is only a very small, almost no, overlap of orbital regions. The orbital hybridization effect is very weak, which further indicates that CH_4_ and H2 are only physically adsorbed on the surface of Cr-SnP_3_.

In Figure 7(f1,f2), the TDOS curves of C_2_H_6_/Cr-SnP_3_ are generally shifted to the right due to the weak orbital hybridization between C and Cr atoms, which leads to a small overlap of PDOS peaks in the range of −15 to −5 eV, but this weak orbital interaction is not sufficient to cause significant charge transfer or significant state changes.

### 3.3. Differential Charge Density Analysis

**Figure 8:**
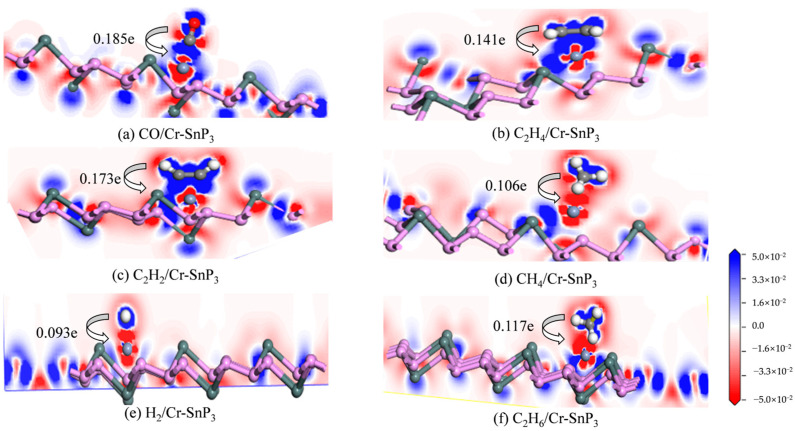
Differential charge density distributions of (**a**) CO, (**b**) C_2_H_4_, (**c**) C_2_H_2_, (**d**) CH_4_, (**e**) H_2_, and (**f**) C_2_H_6_ molecules on the Cr-SnP_3_ monolayer.

The differential charge density of the CO, C_2_H_4_, C_2_H_2_, CH_4_, H_2_, and C_2_H_6_ gas molecule adsorption models (red represents electron aggregation; blue represents electron depletion) are shown in Figure 8. The colour map ranges from −0.05 to 0.05 e/Å. This suggests that during the adsorption process, electrons are moved from the gas molecules to the Cr-SnP3 monolayer. In Figure 8a–c, the red electron concentration region of Cr atoms is almost directly connected to the blue electron depletion region of the gas molecules, which demonstrates that the electron exchange between gas molecules and the Cr-SnP_3_ monolayer is very close, and the Q_t_ of the three gases is considerable. Therefore, the interaction between CO, C_2_H_2_, C_2_H_4_, and Cr-SnP_3_ monolayers is chemical adsorption. The charge transfer of CH_4_, H_2_, and C_2_H_6_ in Figure 8d–f is relatively small, so it can be assumed that the interactions between them are dominated by van der Waals forces. Differential charge density analysis verified the adsorption of the Cr-SnP_3_ monolayer on six gas molecules (CO, C_2_H_4_, C_2_H_2_, CH_4_, H_2_, and C_2_H_6_) from the side.

### 3.4. HOMO-LUMO Energy Gaps

**Figure 9:**
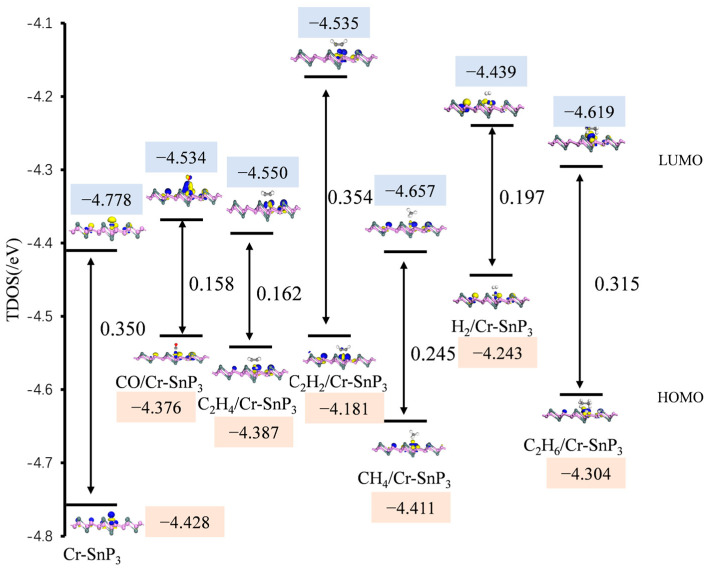
HOMO and LUMO distributions of Cr-SnP_3_ monolayer and six gas-molecule-adsorption systems.

The HOMO, LUMO, and E_g_ of the intrinsic Cr-SnP_3_ and the gas/Cr-SnP_3_ system are shown in Figure 9. The energy gap (E_g_) can be used to analyse the conductivity of the system [23]. For the resistive gas sensor, the change in conductivity (σ) is an important standard to evaluate the applicability of the sensor when detecting gas [24].

It can be seen from Figure 9 that the energy gap of the C_2_H_2_ adsorption system almost did not change much, but the energy gap in the CO, C_2_H_4_, CH_4_, H_2_, and C_2_H_6_ adsorption systems decreased significantly, and the reduction ranges were 54.8%, 53.7%, 30%, 43.7%, and 10%, respectively. The E_g_ of the system decreases after adsorption, suggesting that the system’s conductivity is growing. As the Cr-SnP_3_ monolayer has a poor adsorption effect on CH_4_, H_2_, and C_2_H_6_, the Cr-SnP_3_ monolayer may be employed as a gas-sensitive sensor material for detecting CO and C_2_H_4_.

### 3.5. Recovery Time

Recovery time is an important metric that has to be considered in practical gas sensor requirements, as a too-small or too-large recovery time is not ideal for gas sensors [25]. A longer recovery time will make the sensor more difficult to remove [26]. The recovery time *τ* and the adsorption energy E_ads_ can be related, and can be calculated by Equation (5).

(5) $$\tau =A^{-1}e^{\frac{{-E}_{B}}{K_{B}T}}$$

where *A* is the attempt frequency (10^12^ s^−1^); *E_B_* is equal in magnitude to the adsorption energy [25]; *K_B_* is the Boltzmann constant (8.62 × 10^−5^ eVK^−1^); T is the Kelvin temperature.

In this paper, the recovery time of 298 K, 348 K, 398 K, and 448 K at four different temperatures was calculated. The recovery time of six gases is shown in Table 3. It can be seen that CH_4_, H_2_, and C_2_H_6_ have a short recovery time due to their low adsorption energy. The recovery rate is extremely fast in the temperature range of 298 K~448 K, which takes less than 1s. It is verified from the side that the adsorption of CH_4_, H_2_, and C_2_H_6_ on the surface of Cr-SnP_3_ is extremely unstable. The Cr-SnP_3_ monolayer is not appropriate for detecting these three gases, and it is also impossible to achieve the effective removal of these three gases.

**Figure 10:**
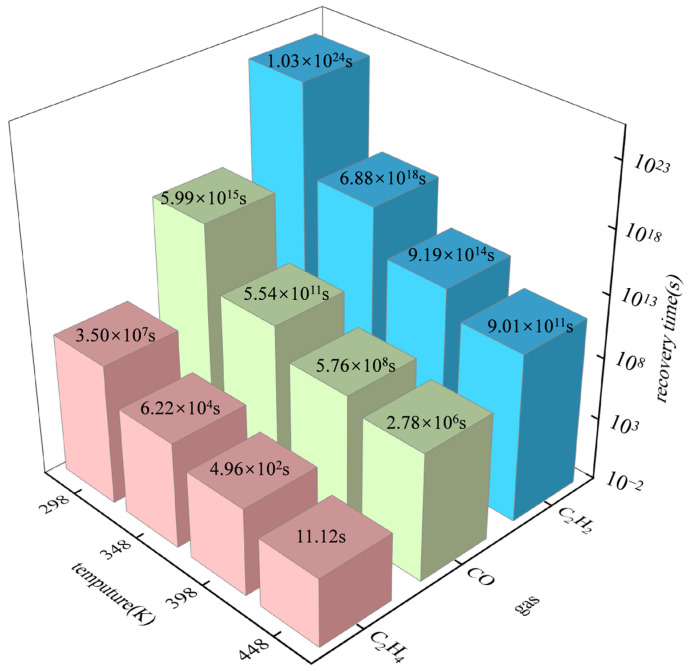
Recovery time of C_2_H_2_, CO, and C_2_H_4_.

Since CH_4_, H_2_, and C_2_H_6_ molecules have been repeatedly confirmed to have no good adsorption characteristics on the Cr-SnP_3_ monolayer, only the recovery times of CO, C_2_H_4_, and C_2_H_2_ are presented. A recovery time histogram of the three gases is shown in Figure 10. Combined with the chart analysis, it was determined that the recovery time of CO and C_2_H_2_ gases was very long in the temperature range of 298 K~448 K. In the normal temperature environment, the recovery time was as long as several decades, which demonstrates that CO and C_2_H_2_ may be adsorbed by the Cr-SnP_3_ monolayer with excellent effectiveness. Nevertheless, for C_2_H_4_ gas, the recovery time decreases to 496 s at 398 K and 11.12 s at 448 K, which indicates that the recovery time of the C_2_H_4_ gas is ideal, so Cr-SnP_3_ can be quickly recycled. The conductivity changes greatly, which meets the requirements of actual gas sensors. However, the temperature should not be too high; otherwise, the recovery time is too short to detect C_2_H_4_. Following a comprehensive analysis, the Cr-SnP_3_ monolayer can be considered as an adsorbent for CO and C_2_H_2_ gases, and it can be used as a resistive gas sensor for C_2_H_4_ at about 398 K.

### 3.6. Comparison of Intrinsic and Doped SnP_3_ Monolayers for the Adsorption of Dissolved Gases in Six Oils

**Figure 11:**
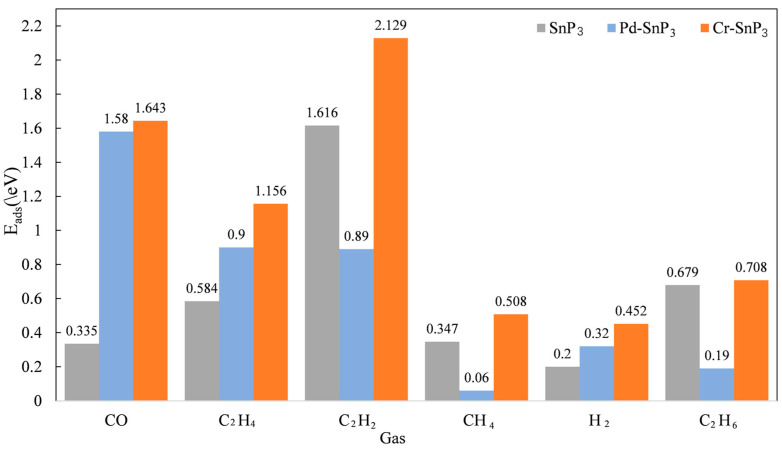
Comparison of the adsorption energy of SnP_3_, Pd-SnP_3_, and Cr-SnP_3_ monolayers on dissolved gas in oil.

To further explore the possibility of using SnP_3_ monolayers as a gas-sensitive sensor material for application in the power industry, the absolute values of the adsorption energy of SnP_3_, Pd-SnP_3_ [20], and Cr-SnP_3_ monolayers on the six gases are compared. The comparison results are shown in Figure 11. Intrinsic SnP_3_ has large adsorption energy only for C_2_H_2_ and adsorption energy is poor for other gases. The addition of Pd or Cr resulted in a significant increase in the adsorption of six gases, especially CO, C_2_H_4_, and C_2_H_2_. It can be observed from the figure that Cr-SnP_3_ is better adsorbed compared to Pd-SnP_3_. Not only does the Cr-SnP_3_ exhibit a stable adsorption capacity for CO and C_2_H_2_, it has both an adsorption effect and an excellent recovery time for C_2_H_4_.

Cr and Pd are both transition metals, but Cr is more widely used and has a higher output, making it more readily available. The metal Cr has a significant cost advantage. Therefore, the addition of Cr is an effective means for SnP_3_ to adsorb and detect gases.

## 4. Conclusions

This work offers a comprehensive investigation of the adsorption process of six gases (CO, C_2_H_4,_ C_2_H_2_, CH_4_, H_2_, and C_2_H_6_) on the Cr-SnP_3_ monolayer. The conclusions are as follows:

(1) Compared with the intrinsic SnP_3_ monolayer, the adsorption effect of a Cr-SnP_3_ monolayer on six dissolved gases (CO, C_2_H_4_, C_2_H_2_, CH_4_, H_2_, and C_2_H_6_) in oil was significantly enhanced. The growth rates of the adsorption energy were 390.4%, 97.9%, 31.7%, 46.3%, 126.1%, and 4.2%, respectively.
(2) The adsorption energies of CO, C_2_H_4_, C_2_H_2_, CH_4_, H_2_, and C_2_H_6_ on the Cr-SnP_3_ monolayer are −1.643, −1.156, −2.129, −0.508, −0.452, and −0.708 eV, respectively. The adsorption strengths were C_2_H_2_ > CO > C_2_H_4_ > C_2_H_6_ > CH_4_ > H_2_. The gas molecules of C_2_H_2_, CO, and C_2_H_4_ undergo chemical adsorption, and the gas molecules of C_2_H_6_, CH_4_, and H_2_ undergo physical adsorption.
(3) Considering the energy gap and recovery time, the Cr-SnP_3_ monolayer can be considered a high-performance adsorbent for CO and C_2_H_2_ gases and a resistive gas sensor for C_2_H_4_.

In conclusion, SnP_3_ is a promising gas-sensitive and adsorbent material. We hope that this paper will strongly promote the gas-sensing application of the Cr-SnP_3_ and contribute to state-monitoring technology for oil-immersed power equipment in the future. This paper mainly focused on a theoretical study of the ideal case. We will be committed to improving this theoretical research with experimental data in the future.

## Figures and Tables

**Table 1:** Structural parameters and η of four doping systems.

System	E_b_ (eV)	Q_t_ (e)	η (eV)
T_P_	−3.123	0.087	0.0199
T_Sn_	−4.600	0.210	0.1752
H_pSn_	−3.653	0.101	0.1414
H_P_	−4.143	0.151	0.1541

**Table 2:** Adsorption parameters of CO, C_2_H_4_, C_2_H_2_, CH_4_, H_2_, and C_2_H_6_ on SnP_3_ and Cr-SnP_3_.

System	E_ads_ (eV)	Q_t_ (e)	d (Å)
CO/SnP_3_	−0.335	0.009	2.849
C_2_H_4_/SnP_3_	−0.584	0.016	3.008
C_2_H_2_/SnP_3_	−1.616	−0.200	2.123
CH_4_/SnP_3_	−0.347	−0.020	3.310
H_2_/SnP_3_	−0.200	0.0007	2.500
C_2_H_6_/SnP_3_	−0.679	0.005	3.325
CO/Cr-SnP_3_	−1.643	0.185	1.939 (C-Cr)
C_2_H_4_/Cr-SnP_3_	−1.156	0.141	2.070 (C-Cr)
C_2_H_2_/Cr-SnP_3_	−2.129	0.173	1.896 (C-Cr)
CH_4_/Cr-SnP_3_	−0.508	0.106	2.465 (C-Cr)
H_2_/Cr-SnP_3_	−0.452	0.093	1.916 (H-Cr)
C_2_H_6_/Cr-SnP_3_	−0.708	0.117	2.254 (C-Cr)

**Table 3:** Recovery time of six gases at different temperatures.

Gas	Recovery Time/s			
	298 K	348 K	398 K	448 K
CO	5.99 × 10^15^	5.54 × 10^11^	5.76 × 10^8^	2.78 × 10^6^
C_2_H_4_	3.50 × 10^7^	6.22 × 10^4^	4.96 × 10^2^	11.12
C_2_H_2_	1.03 × 10^24^	6.88 × 10^18^	9.19 × 10^14^	9.01 × 10^11^
CH_4_	3.87 × 10^−4^	2.69 × 10^−5^	2.38 × 10^−6^	5.44 × 10^−7^
H_2_	4.06 × 10^−5^	3.27 × 10^−6^	4.97 × 10^−7^	1.15 × 10^−7^
C_2_H_6_	6.83 × 10^−1^	1.41 × 10^−2^	5.59 × 10^−4^	7.45 × 10^−5^

## Data Availability

The original contributions presented in the study are included in the article, further inquiries can be directed to the corresponding author.
